# Whole-body muscle magnetic resonance imaging in inflammatory myopathy with mitochondrial pathology

**DOI:** 10.3389/fneur.2024.1386293

**Published:** 2024-04-23

**Authors:** Wagner Cid Palmeira Cavalcante, André Macedo Serafim da Silva, Rodrigo de Holanda Mendonça, Cristiane de Araújo Martins Moreno, Bruna Moreira de Souza Proença, Júlio Brandão Guimarães, Alípio Gomes Ormond Filho, Edmar Zanoteli

**Affiliations:** ^1^Department of Neurology, Faculdade de Medicina da Universidade de São Paulo (FMUSP), São Paulo, Brazil; ^2^Department of Musculoskeletal Radiology, Grupo Fleury Medicina e Saúde, São Paulo, Brazil

**Keywords:** inclusion body myositis, polymyositis, inflammatory myopathy, magnetic resonance imaging, mitochondria

## Abstract

**Introduction:**

Inflammatory myopathy with mitochondrial pathology (IM-Mito) is a rare condition described in a few case series, and it is not clear whether it is a specific disease or a variant of Inclusion Body Myositis (IBM). Radiological data of IM-Mito patients has only been evaluated in one study.

**Aim:**

To analyze whole-body muscle magnetic resonance imaging (MRI) features in patients with IM-Mito compared with individuals with IBM.

**Methods:**

Fourteen IM-Mito and ten IBM patients were included. IM-Mito was defined by endomysial inflammatory infiltrate, presence of at least 1% of Cytochrome C Oxidase negative fibers, and absence of rimmed vacuoles in muscle biopsy; and IBM was defined by the presence of dystrophic muscular abnormalities, endomysial inflammatory infiltrate, and rimmed vacuoles. Patients underwent clinical evaluation and whole-body muscle MRI to determine the presence of edema, and fatty infiltration in various muscles.

**Results:**

Muscle imaging abnormalities were asymmetric in most patients with IM-Mito and IBM. Muscles with the highest average degree of fatty infiltration in both conditions were the quadriceps and medial gastrocnemius. Most patients with IM-Mito and IBM showed imaging patterns of rectus femoris relatively spared compared to other quadriceps muscles. The flexor digitorum profundus was the most affected muscle of the upper limbs in both IBM and IM-Mito.

**Discussion:**

Although the results suggest some similarities in muscle imaging features between IM-Mito and IBM, there remains uncertainty whether these two conditions are part of the same clinical spectrum.

## Introduction

Mitochondrial changes in muscle histology, such as the presence of cytochrome C oxidase negative (COX-) fibers, have been described in varying degrees in inflammatory myopathies (IM) (1, 2). In the last years, a peculiar form of inflammatory myopathy with mitochondrial pathology (IM-Mito) has been described in some case series, including a few dozen patients, but with unknown precise prevalence and incidence (3–9). The original description of this disorder was made in 1997 by Blume et al. (3), who reported ten patients with muscle histology typical of polymyositis, but with excessive COX-fibers and poor response to immunosuppressive therapy. In following series, these cases were defined according to muscle histological findings that demonstrate a combination of inflammatory infiltrate of CD8+ T lymphocytes along with mitochondrial changes such as the presence of more than 1–3% COX-fibers (3–9). Previously published clinical reports of IM-Mito generally demonstrate that these patients have relatively higher age than those with autoimmune IM, female predominance, heterogeneous clinical phenotype, and variable response to immunosuppression (3–9). Given these epidemiological, clinical and pathological characteristics, it remains unclear whether IM-Mito is an atypical form of IM or a spectrum of sporadic inclusion body myositis (IBM).

Muscle magnetic resonance imaging (MRI) has become an essential complementary test in the diagnosis and follow-up of patients with myopathies (10). Radiological features of muscle MRI that may appear in patients with IM are edema in the acute phase and atrophy with fatty infiltration in later stages (11, 12). The pattern and distribution of abnormalities seen on muscle MRI may vary depending on the subtype of IM, providing clues to specific diagnoses. The most common muscle MRI imaging finding in polymyositis consists of bilateral and symmetrical edema in muscles of the pelvic girdle and thighs (13). In contrast, patients with IBM have muscle MRI findings with a pattern of muscle atrophy and fatty infiltration more evident than edema, primarily affecting the anterior compartments of both the thighs and forearms (14, 15). In the only publication addressing radiological features in patients with IM-Mito, Zierer et al. (16) concluded that MRI findings in patients with IM-Mito relevantly differed from IBM.

Given the uncertainties regarding IM-Mito and the scarce radiological data available in medical literature, we designed a study to compare muscle MRI characteristics between patients with IM-Mito and IBM.

## Materials and methods

**Figure 1:**
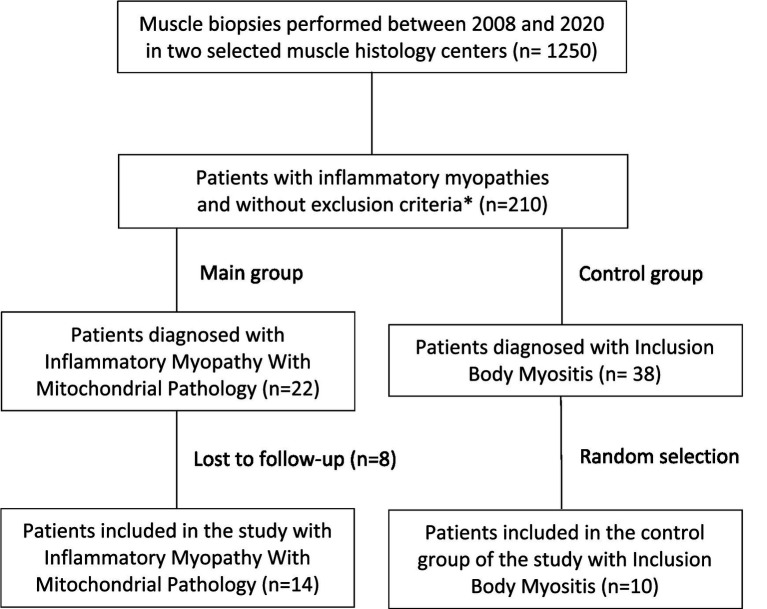
Flowchart of patient selection. *Exclusion criteria for the study: claustrophobia, cognitive impairment, or genetically determined myopathy (familial IBM).

We conducted an observational study of patients with IM-Mito and IBM previously diagnosed in databases of two muscle pathology centers from 2008 to 2020. In the above-mentioned period, 22 patients with IM-Mito and 38 patients with IBM were identified according to histological criteria. After excluding individuals with claustrophobia, cognitive impairment, genetically determined myopathy (familial IBM), or lost follow-up, 14 patients with IM-Mito were included in the study after voluntarily agreeing to participate. Because study funding included a limited amount of 24 whole-body muscle MRI exams, we opted to include an additional randomly selected 10 participants, with IBM designated as a control group (Figure 1). The institutional ethics committee approved the study (Protocol number 3.460.324/CAAE number 93788218.0.3001.0068).

### Clinical data

Clinical data collected included age, gender, family history, comorbidities, current and previous medications, use of immunosuppressants, clinical response to immunosuppressants, age of symptoms onset, progression of symptoms, distribution of muscle weakness, gait impairment, dysphagia, fatigue, myalgia, and cramps. Participants underwent a detailed neurological exam assessing strength in several muscle groups, deep tendon reflexes, muscle trophism, muscle tone, gait, sensory abnormalities, and cranial nerves. Quantitative assessment of muscle strength was obtained using the Medical Research Council (MRC) five-point scale (Medical Research Council 1968). Regarding diagnostic criteria for IBM, patients were assessed according to the European Neuromuscular Center and Lloyd criteria (17, 18).

### Muscle biopsy

Both groups of patients were defined according to muscle histopathological criteria. Muscle biopsies were performed and analyzed by a physician with long experience in muscle pathology (EZ). Muscle biopsies were performed according to institutional protocols in a surgical environment, with local anesthesia, and with muscle fragments taken from the biceps brachii or quadriceps femoris muscles. All specimens were submitted to standard histological staining with hematoxylin–eosin and modified Gomori’s trichrome. In addition, histochemical stains such as ATP4.3, ATP4.6, ATP9.4, Citochorome c oxidase (COX), NADH, and SDH were also performed. Finally, immunohistochemistry evaluations were conducted for MHC-I, CD4, CD8, CD68, p62, and TDP-43. Based on the presence of size variability among muscle fibers, increased endomysial/perimysial connective tissue, proportion of fibers with nuclear centralization and the presence of necrotic fibers, the samples were classified as normal (−), or dystrophic (+, mild; ++; moderate; +++, severe). Additional histological findings included the presence of inflammatory reaction, rimmed vacuoles, and mitochondrial abnormalities (ragged-red fibers and COX-fibers).

Quantification of COX-fibers percentage was performed after counting at least 200 muscle fibers on COX/SDH double histological staining, in a 20× magnification and different sites of the muscle fragments.

The histological definition of IM-Mito was based on the presence of inflammatory infiltrate with predominant CD8+ lymphocytes, 1% or more COX-fibers, and the absence of rimmed vacuoles. IBM was defined based on the presence of inflammatory infiltrate and the presence of rimmed vacuoles with or without the presence of COX-fibers.

### Whole-body MRI

Whole-body magnetic resonance imaging (WBMRI) was performed without sedation and at high magnetic field (1.5 Tesla) equipment. Images were acquired in the axial plane with T1-weighted, Short Tau Inversion Recovery (STIR) and Fast Spin Echo (FSE) sequences. Body and surface coils were used for signal transmission and reception.

Technical parameters used in MRI sequences were described according to the areas analyzed, which were divided into 7 regions: right and left arms (TR = 450 ms; TE = minimum; FOV = 20 cm; thickness = 7.0 mm, gap = 0.5 cm), right and left forearms (TR = 450 ms; TE = minimum; FOV = 16.0 cm; thickness = 7.0 cm; gap = 0.5 cm), pelvis, thighs and legs (TR = 600 ms; TE = minimum; FOV = 35 cm; gap = 1.0 cm). The total approximate examination time was 60 min.

Evaluated muscles included: tensor fascia lata, iliopsoas, gluteus minimus, gluteus maximus, gluteus medius, vastus medialis, vastus intermedius, vastus lateralis, rectus femoris, semitendinosus, biceps femoris, semimembranosus, adductor magnus, adductor longus, gracilis, sartorius, medial gastrocnemius, lateral gastrocnemius, soleus, tibialis anterior, tibialis posterior, extensor digitorum longus, fibularis, deltoid, biceps brachii, triceps, brachioradialis, extensor carpi ulnaris, extensor carpi radialis, extensor digitorum communis, pronator quadratus, supinator, flexor carpi ulnaris, flexor carpi radialis, flexor digitorum superficialis and flexor digitorum profundus.

Muscle images were analyzed by two radiologists specialized in musculoskeletal diseases (JBG, AGOF) without knowledge of clinical data. Images were independently evaluated, and the result was obtained through a consensus between both specialists.

Muscle fatty infiltration was defined on T1 weighted MRI sequences, and it was graded according to a semiquantitative 4-point visual scale (19): grade 1 - normal (no fatty infiltration); Grade 2 - mild (less than 30% of fatty infiltration); Grade 3 - moderate (more than 30% and less than 60% of fatty infiltration); Grade 4 - severe (more than 60% of fatty infiltration). The edema pattern was analyzed using STIR-weighted MRI sequences and classified as present or absent (20).

Symmetry of radiological involvement in WBMRI was assessed by analyzing all four limbs simultaneously, comparing muscle involvement and severity between the left and right sides. Asymmetric imaging involvement was defined when there was a difference in the presence of edema or degree of fatty infiltration between the right and left sides in at least one muscle.

We used a graphical technique described as a heatmap to present a visual interface of muscles evaluated in each patient, thus allowing a quick illustration of the imaging appearance of IM-Mito and IBM (21). The heatmaps were built according to the average degree of fatty infiltration on each muscle in both groups of IM-Mito and IBM.

### Statistical analysis

The sample was divided into two groups according to the diagnosis of IM-Mito or IBM. Group characteristics were described using absolute and relative frequencies for qualitative variables and means and standard deviations for quantitative variables. Comparisons between groups were performed by Wilcoxon’s rank sum test for quantitative variables or chi-square and Fisher’s exact test for qualitative variables. Statistical analyses were performed in R software, considering a *p*-value below 0.05 as statistically significant.

## Results

Fourteen patients with IM-Mito and ten individuals with IBM were evaluated. Table 1 presents the clinical data comparison between patients with IM-Mito and IBM. Regarding physical examination, the two groups had some significant distinct clinical findings (Table 2). Muscle histological data are presented in Table 3.

**Table 1:** Clinical data in patients with inflammatory myopathy with mitochondrial pathology (IM-Mito) and inclusion body myositis (IBM).

Features	IM-Mito, *N* = 14^a^	IBM, *N* = 10^a^	*p* value^b^
Current age (years)	56,9 (10.4)	69.1 (8.6)	0.011
Age of symptom onset (years)	50.1 (11.5)	60.8 (7.6)	0.018
Sex			0.013
Female	10 (71.4%)	2 (20.0%)	
Male	4 (28.6%)	8 (80.0%)	
Cancer	3 (21.4%)	1 (10.0%)	0.615
Autoimmune disease	3 (21.4%)	2 (20.0%)	>0.999
Disease duration (months)	81.9 (57.2)	87.2 (35.7)	0.639
Muscle weakness	12 (85.7%)	10 (100.0%)	0.493
Cramps	5 (35.7%)	2 (20.0%)	0.653
Myalgia	10 (71.4%)	2 (20.0%)	0.013
Fatigue	11 (78.6%)	4 (40.0%)	0.092
Gait impairment	3 (21.4%)	6 (60.0%)	0.092
Dysphagia	7 (50.0%)	5 (50.0%)	>0.999
Early falls	5 (35.7%)	9 (90.0%)	0.013
Immunosuppressor use	10 (71.4%)	5 (50.0%)	0.403
Subjective improvement with immunosuppressors	3 (30.0%)	0 (0.0%)	0.505
Serum creatine kinase (U/L)	1,038.6 (821.4)	1,980.6 (1,733.7)	0.172
European Neuromuscular Center probable or definite diagnostic criteria for IBM	3 (21.4%)	9 (90.0%)	<0.001
Lloyd criteria compatible with IBM	7 (50.0%)	10 (100.0%)	0.019

a Mean (standard deviation); *n* (%).

b Wilcoxon’s rank sum test; chi-square; Fisher’s exact test.

**Table 2:** Neurological examination and clinical phenotypes in patients with inflammatory myopathy with mitochondrial pathology (IM-Mito) and inclusion body myositis (IBM).

Features	IM-Mito, *N* = 14^a^	IBM, *N* = 10^a^	*p* value^b^
Finger flexor muscle weakness	5 (35.7%)	9 (90.0%)	0.013
Quadriceps muscle weakness	9 (64.3%)	10 (100.0%)	0.053
Greater quadriceps weakness compared with ileopsoas muscle	0 (0.0%)	5 (50.0%)	0.006
Finger flexor muscles weaker than deltoid muscle	4 (28.5%)	9(90%)	0.004
Quadriceps or finger flexor muscles atrophy	5 (35.7%)	10 (100.0%)	0.002
Asymmetric muscle weakness in physical exam	3 (21.4%)	8 (80.0%)	0.011
Fist sign	3 (21.4%)	8 (80.0%)	0.011
Oligosymptomatic phenotype	4 (28.6%)	0 (0.0%)	0.114
Proximal muscle weakness phenotype	5 (35.7%)	1 (10.0%)	0.340
Proeminent quadríceps or finger flexor muscles weakness phenotype	4 (28.6%)	9 (90.0%)	0.004
Diffuse muscle weakness phenotype	1 (7.1%)	0 (0.0%)	>0.999

a *n* (%).

b Chi-square; Fisher’s exact test.

**Table 3:** Histological changes in muscles biopsies of patients with inflammatory myopathies with mitochondrial pathology (IM-Mito) and inclusion body myositis (IBM).

N	Group	Dystrophic pattern	Vacuoles	Inflammation	COX-	CD68/CD8/MHC-I	TDP-43/p62
1	IM-Mito	−	A	++	3%	++/++/+	A/A
2	IM-Mito	+	A	++	1%	++/+/++	P/A
3	IM-Mito	−	A	+	1%	++/+/+	A/A
4	IM-Mito	+	A	++	10%	++/+/++	A/A
5	IM-Mito	−	A	+	1%	+/+/+	A/A
6	IM-Mito	−	A	++	1%	+/+/+	A/P
10	IM-Mito	+	A	++	1%	++/+/++	A/A
16	IM-Mito	−	A	+	1%	+/+/+++	A/A
17	IM-Mito	+	A	+++	1%	++/++/+++	A/P
18	IM-Mito	−	A	+	1%	+/+/++	A/A
20	IM-Mito	+	A	++	2%	+/+/+++	A/A
22	IM-Mito	+	A	++	3%	++/++/+++	A/P
23	IM-Mito	+	A	+	10%	+/+/+++	A/A
24	IM-Mito	+	A	+	5%	++/+/++	A/A
7	IBM	+++	P	+++	10%	+++/+++/++	P/P
8	IBM	+	P	++	5%	++/++/+++	A/A
9	IBM	+	P	++	1%	+++/+++/++	P/P
11	IBM	++	P	+	1%	+/+/+	P/P
12	IBM	+	P	+++	2%	+++/+++/++	A/P
13	IBM	++	P	++	1%	++/++/+++	P/P
14	IBM	+	P	++	1%	++/++/++	A/P
15	IBM	++	P	++	5%	++/++/+++	P/P
19	IBM	+	P	++	1%	++/++/++	A/A
21	IBM	+	P	++	7%	++/++/++	A/A

A, absent; P, present; −, no change; +, mild; ++, moderate; +++, severe.

### Whole-body muscle MRI

Among muscle groups evaluated by WBMRI in patients with IM-Mito and IBM, the presence of fatty infiltration and edema, and the mean degree of fatty infiltration according to Mercuri score were similar in most muscles (Tables 4–6). On the other hand, patients with IBM had a higher prevalence of fatty infiltration in the biceps femoris, semimembranosus, gracilis, tensor fascia lata, and lateral gastrocnemius muscles compared to individuals with IM-Mito. Regarding muscle edema, only the vastus medialis muscle demonstrated a more significant presence of edema in patients with IBM than in those with IM-Mito (100% vs. 57.1%, respectively, *p* = 0.01).

**Table 4:** Presence of muscle fatty infiltration evaluated by whole-body muscle magnetic resonance imaging in patients with inflammatory myopathy with mitochondrial pathology (IM-Mito) and inclusion body myositis (IBM).

Muscle	IM-Mito, *N* = 14^a^	IBM, *N* = 10^a^	*p* value^b^
Vastus lateralis	14 (100%)	10 (100%)	1,0
Vastus medialis	11 (78,5%)	8 (80%)	0,93
Vastus intermedius	9 (64,2%)	8 (80%)	0,40
Rectus femoris	5 (35,7%)	6 (60%)	0,23
Biceps femoris	5 (35,7%)	8 (80%)	0,03
Semimembranosus	9 (64,2%)	10 (100%)	0,03
Semitendinosus	6 (42,8%)	8 (80%)	0,06
Gracilis	4 (28,5%)	8 (80%)	0,03
Sartorius	6 (42,8%)	7 (70%)	0,18
Tensor fascia lata	6 (42,8%)	9 (90%)	0,01
Adductor	6 (42,8%)	8 (80%)	0,06
Iliopsoas	2 (14,2%)	1 (10%)	1,0
Gluteus maximus	2 (14,2%)	3 (30%)	0,61
Gluteus medius	4 (28,5%)	2 (20%)	1,0
Gluteus minimus	8 (57,1%)	10 (100%)	0,16
Medial gastrocnemius	12 (85,7%)	10 (100%)	0,21
Lateral gastrocnemius	5 (37,5%)	8 (80%)	0,03
Soleus	8 (57,1%)	9 (90%)	0,08
Tibialis anterior	6 (42,8%)	7 (70%)	0,18
Tibialis posterior	6 (42,8%)	5 (50%)	0,72
Extensor digitorum longus	4 (28,5%)	6 (60%)	0,21
Fibularis	6 (42,8%)	7 (70%)	0,18
Biceps brachii	5 (37,5%)	5 (50%)	0,48
Triceps	3 (21,4%)	3 (30%)	0,66
Deltoid	3 (21,4%)	3 (30%)	0,66
Flexor carpi ulnaris	0 (0%)	0 (0%)	1,0
Flexor carpi radialis	0 (0%)	0 (0%)	1,0
Finger flexors (flexor digitorum profundus)	9 (64,2%)	9 (90%)	0,15
Brachioradialis	0 (0%)	0 (0%)	1,0
Carpal extensors	0 (0%)	0 (0%)	1,0
Common extensor of the fingers	2 (14,2%)	4 (40%)	0,19
Supinator and pronator quadratus	1 (7,1%)	1 (10%)	1,0

a *n* (%).

b Chi-square; Fisher’s exact test.

**Table 5:** Presence of muscle edema evaluated by whole-body muscle magnetic resonance imaging in patients with inflammatory myopathy with mitochondrial pathology (IM-Mito) and inclusion body myositis (IBM).

Muscle	IM-Mito, *N* = 14^a^	IBM, *N* = 10^a^	*p* value^b^
Vastus lateralis	8 (57,1%)	9 (90%)	0,08
Vastus medialis	8 (57,1%)	10 (100%)	0,01
Vastus intermedius	2 (14,2%)	4 (40%)	0,19
Rectus femoris	5 (35,7%)	5 (50%)	0,48
Biceps femoris	5 (35,7%)	5 (50%)	0,48
Semimembranosus	3 (21,4%)	5 (50%)	0,20
Semitendinosus	2 (14,2%)	2 (20%)	1,0
Gracilis	0 (0%)	1 (10%)	0,41
Sartorius	1 (7,1%)	1 (10%)	1,0
Tensor fascia lata	1 (7,1%)	2 (20%)	0,55
Adductor	5 (35,7%)	4 (40%)	1,0
Iliopsoas	2 (14,2%)	0 (0%)	0,49
Gluteus maximus	0 (0%)	0 (0%)	1,0
Gluteus medius	1 (7,1%)	0 (0%)	1,0
Gluteus minimus	1 (7,1%)	3 (30%)	0,27
Medial gastrocnemius	7 (50%)	6 (60%)	0,62
Lateral gastrocnemius	3 (21,4%)	5 (50%)	0,20
Soleus	4 (28,5%)	6 (60%)	0,21
Tibialis anterior	4 (28,5%)	6 (60%)	0,21
Tibialis posterior	3 (21,4%)	3 (30%)	0,66
Extensor digitorum longus	2 (14,2%)	3 (30%)	0,61
Fibularis	4 (28,5%)	3 (30%)	1,0
Biceps brachii	2 (14,2%)	2 (20%)	1,0
Triceps	2 (14,2%)	3 (30%)	0,61
Deltoid	1 (7,1%)	0 (0%)	1,0
Flexor carpi ulnaris	0 (0%)	0 (0%)	1,0
Flexor carpi radialis	0 (0%)	0 (0%)	1,0
Finger flexors (flexor digitorum profundus)	4 (28,5%)	3 (30%)	1,0
Brachioradialis	0 (0%)	0 (0%)	1,0
Carpal extensors	0 (0%)	0 (0%)	1,0
Common extensor of the fingers	2 (14,2%)	2 (20%)	1,0
Supinator and pronator quadratus	0 (0%)	0 (0%)	1,0

a *n* (%).

b Chi-square; Fisher’s exact test.

**Table 6:** Mean degree of fatty infiltration according to Mercuri score in muscles of patients with inflammatory myopathy with mitochondrial pathology (IM-Mito) and inclusion body myositis (IBM).

Muscle	IBM, *N* = 10^a^	IM-Mito, *N* = 14^a^	*p* value^b^
Medial gastrocnemius	3,6 (0,7)	3,0 (1,2)	0,305
Vastus lateralis	3,3 (0,8)	3,3 (0,8)	0,850
Vastus intermedius	3,1 (1,2)	2,4 (1,3)	0,219
Vastus medialis	3,0 (1,1)	2,7 (1,2)	0,629
Semimembranosus	2,8 (0,8)	2,0 (1,1)	0,056
Gluteus minimus	2,7 (0,7)	2,0 (1,1)	0,071
Soleus	2,7 (1,0)	2,0 (1,1)	0,138
Tensor fascia lata	2,6 (1,1)	1,8 (1,1)	0,072
Fibularis	2,5 (1,3)	1,7 (1,0)	0,105
Sartorius	2,5 (1,2)	1,9 (1,2)	0,239
Biceps femoris	2,4 (1,1)	1,9 (1,2)	0,215
Semitendinosus	2,2 (1,1)	1,9 (1,2)	0,386
Gracilis	2,2 (1,0)	1,7 (1,3)	0,095
Tibialis anterior	2,2 (1,0)	1,6 (0,9)	0,113
Finger flexors (flexor digitorum profundus)	2,2 (0,9)	2,1 (1,2)	0,524
Lateral gastrocnemius	2,2 (1,1)	1,9 (1,3)	0,251
Rectus femoris	2,1 (1,1)	1,6 (0,9)	0,210
Adductor	2,1 (0,9)	1,7 (1,0)	0,153
Biceps brachii	1,9 (1,1)	1,6 (1,0)	0,533
Extensor digitorum longus	1,7 (0,7)	1,4 (0,6)	0,207
Tibialis posterior	1,6 (0,7)	1,6 (0,9)	0,767
Gluteus maximus	1,5 (1,0)	1,4 (1,1)	0,482
Triceps	1,4 (0,7)	1,4 (0,8)	0,699
Deltoid	1,4 (0,7)	1,5 (1,1)	0,817
Common extensor of the fingers	1,4 (0,5)	1,3 (0,8)	0,229
Iliopsoas	1,3 (0,9)	1,3 (0,7)	0,878
Gluteus medius	1,3 (0,7)	1,6 (1,1)	0,616
Supinator and pronator quadratus	1,1 (0,3)	1,1 (0,5)	0,903
Flexor carpi ulnaris	1,0 (0,0)	1,0 (0,0)	
Flexor carpi radialis	1,0 (0,0)	1,0 (0,0)	
Brachioradialis	1,0 (0,0)	1,0 (0,0)	
Carpal extensors	1,0 (0,0)	1,0 (0,0)	

a Mean (Standard Deviation).

b Wilcoxon signed-rank test.

**Figure 2:**
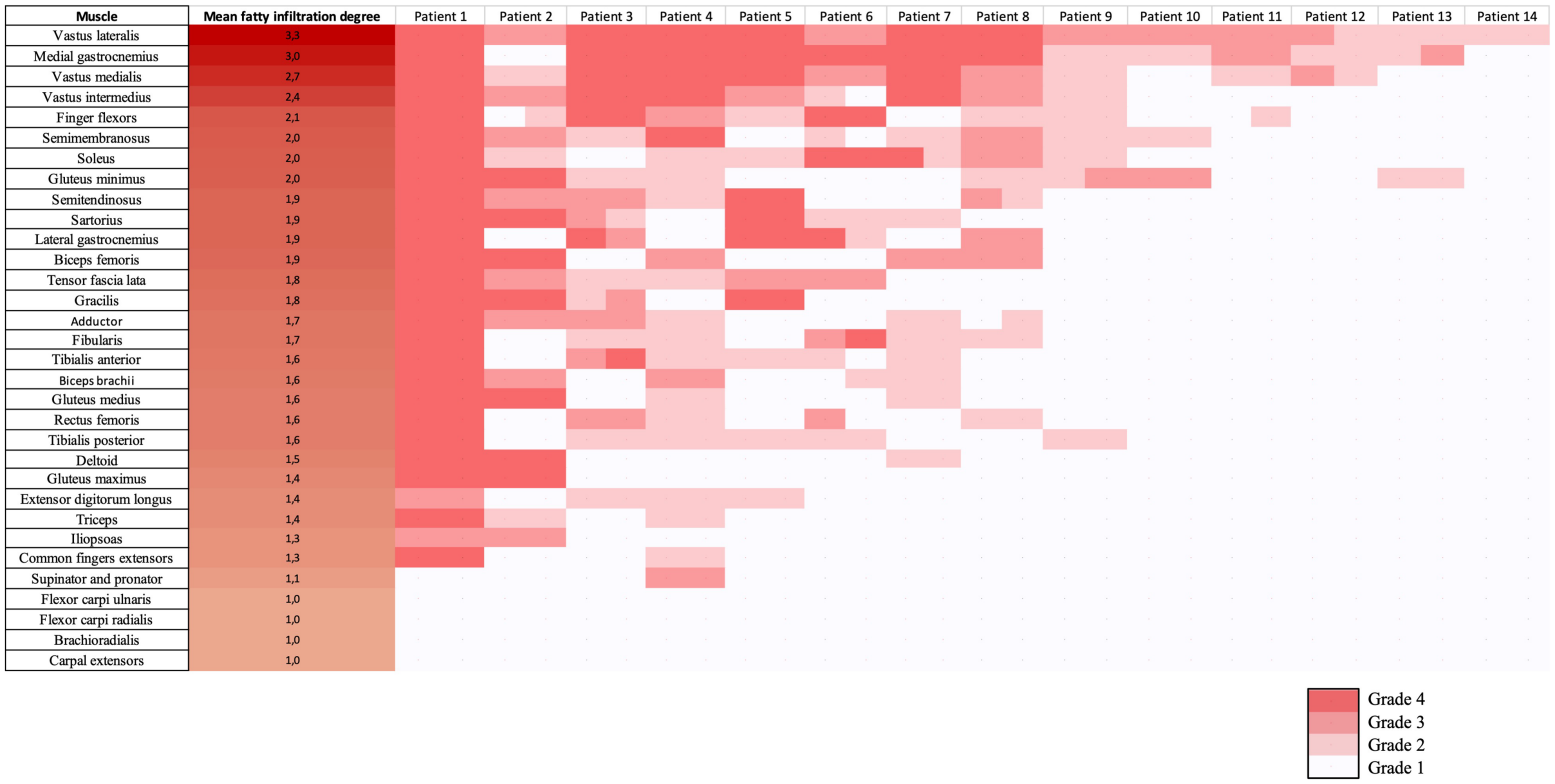
Heatmap evaluation of the mean fatty infiltration degree in whole-body muscle magnetic resonance imaging in patients with inflammatory myopathy with mitochondrial pathology (IM-Mito).

**Figure 3:**
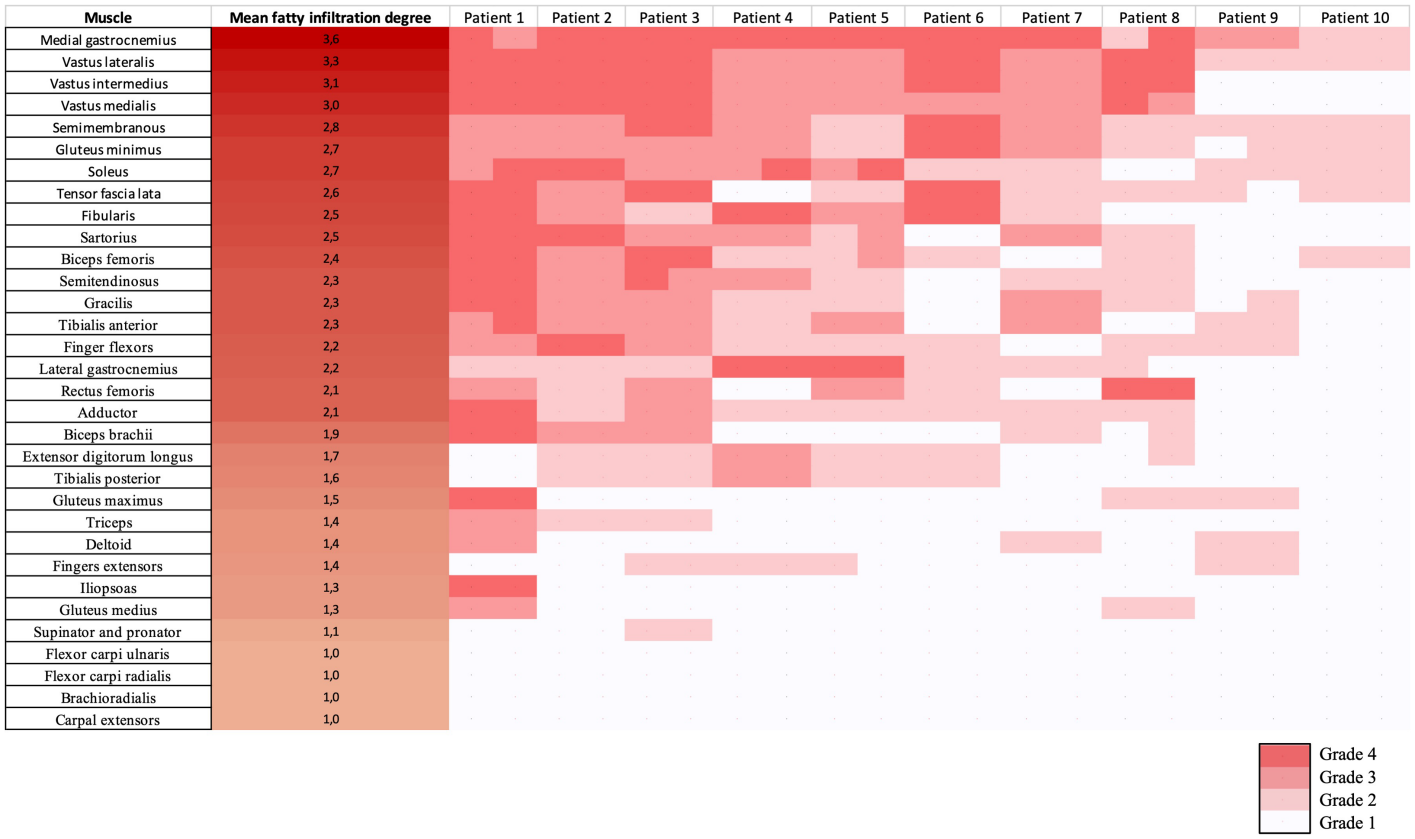
Heatmap evaluation of the mean fatty infiltration degree in whole-body muscle magnetic resonance imaging in patients with inclusion body myositis (IBM).

**Figure 4:**
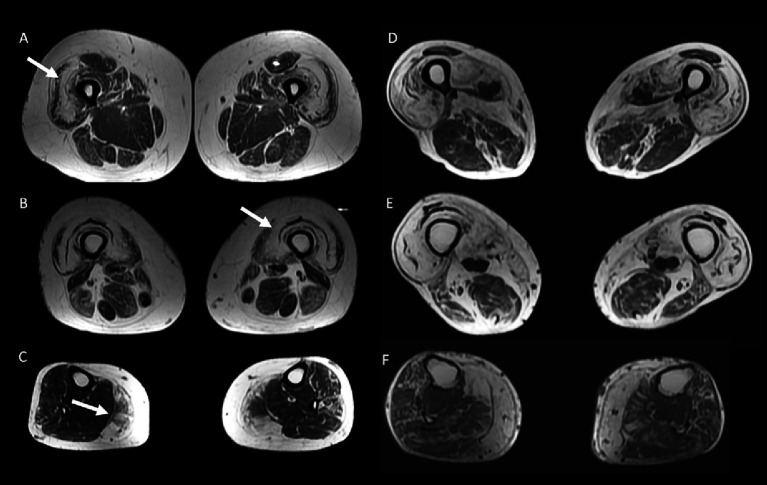
50 year-old female with inflammatory myopathy with mitochondrial pathology (IM-Mito) **(A–C)** presents axial fast spin-echo T1-weighted Whole-Body Muscle Magnetic Resonance Imaging (WBMRI) showing moderate-severe fatty infiltration in quadriceps muscle with relatively spared rectus femoris muscle compared to other quadriceps muscles **(A,B)** and severe fatty infiltration in medial gastrocnemius muscle **(C)**. Axial fast spin-echo T1-weighted WBMRI in a 78 year-old male with inclusion body myositis **(D–F)** shows similar imaging appearance compared to IM-Mito shown in **(A–C)**.

Heatmap evaluation showed that muscles with the highest mean degree of fatty infiltration in WBMRI were quadriceps and medial gastrocnemius in both groups of patients (Figures 2–4). There was no statistically significant difference in the mean degree of fatty infiltration of the various muscles evaluated in patients with IM-Mito and IBM (Table 6). We highlight a trend for patients with IBM to have a higher mean number of muscles with fatty infiltration when compared with IM-Mito (37.9 and 24.6, respectively, *p* = 0.05).

**Figure 5:**
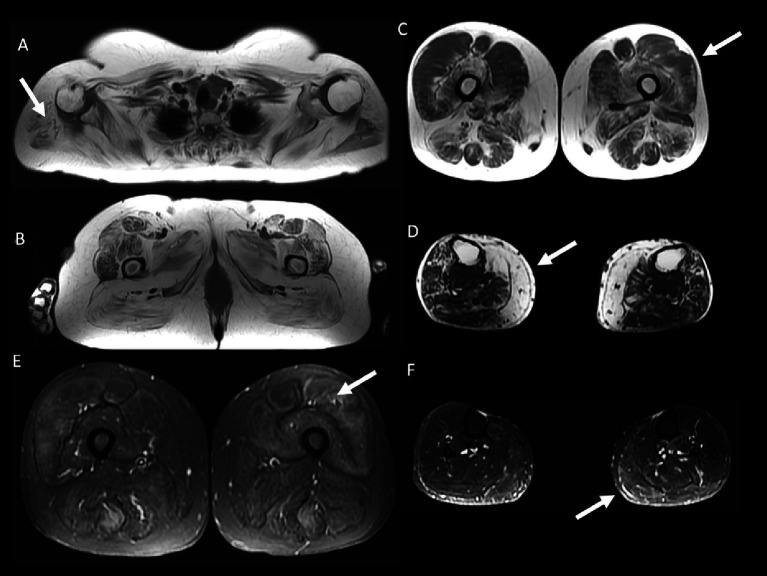
59 year-old female with inflammatory myopathy with mitochondrial pathology (IM-Mito). Axial fast spin-echo T1-weighted Whole-Body Muscle Magnetic Resonance Imaging (WBMRI) **(A–D)** showing severe fatty infiltration in the shoulder girdle muscles **(A)**, moderate-severe fatty infiltration in quadriceps, gluteus and adductors muscles **(B)**, moderate fatty infiltration in quadriceps muscle with relatively spared rectus femoris muscle compared to other quadriceps muscles **(C)** and severe fatty infiltration in the medial gastrocnemius muscle **(D)**. Axial STIR WBMRI shows quadriceps muscle edema **(E)** and gastrocnemius muscle edema **(F)**.

**Figure 6:**
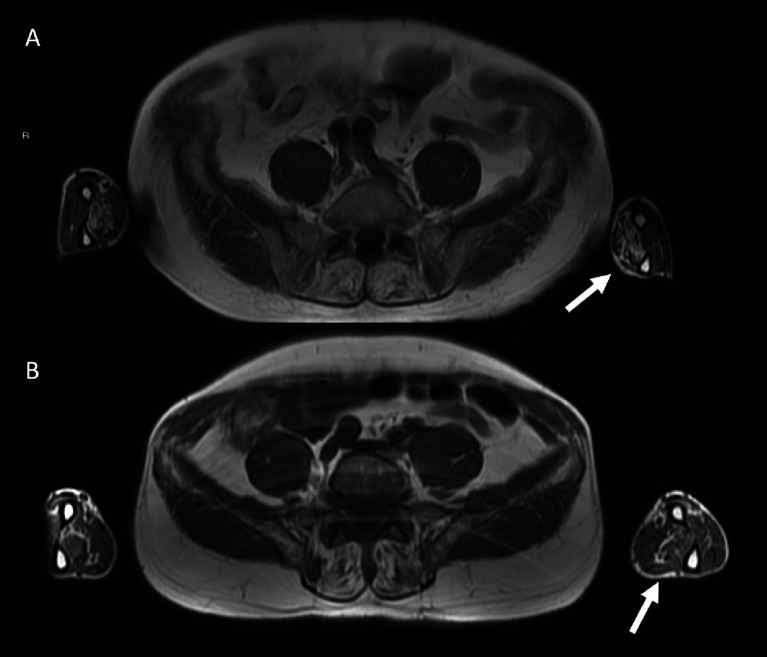
Axial fast spin-echo T1-weighted Whole-Body Muscle Magnetic Resonance Imaging (WBMRI) in a 78 year-old male with inclusion body myositis (IBM) shows moderate-severe fatty infiltration in the flexor digitorum profundus muscles bilaterally **(A)**. A 62 year-old male with inflammatory myopathy with mitochondrial pathology (IM-Mito) shows similar imaging appearance with moderate fatty infiltration in the flexor digitorum profundus muscles bilaterally in axial fast spin-echo T1-weighted WBMRI **(B)**.

Other general radiological findings are shown in Table 7. Muscle edema in at least one muscle was found in most patients with IM-Mito and IBM (Figure 5), but there was no significant difference between the two groups. The mean number of muscles with edema per patient was also similar between the two groups. Asymmetry was observed in most study participants; however, there was no statistically significant difference between patients with IM-Mito and IBM. Most participants with IM-Mito and IBM showed relatively spared rectus femoris muscle compared to other quadriceps muscles (Figure 4). Nevertheless, no statistically relevant difference was found between the groups. The most compromised upper limb muscle by edema or fatty infiltration in patients with IM-Mito and IBM was the flexor digitorum profundus (Figure 6). Although not statistically significant, there was a trend towards greater radiological involvement of these muscles in individuals with IBM compared to those with IM-Mito (100% vs. 64.3%, respectively, *p* = 0.05).

**Table 7:** General radiological features evaluated by whole-body muscle magnetic resonance imaging in patients with inflammatory myopathy with mitochondrial pathology (IM-Mito) and inclusion body myositis (IBM).

Radiological feature	IM-Mito, *N* = 14^a^	IBM, *N* = 10^a^	*p* value^b^
Muscular edema in at least one muscle	12 (85.7%)	10 (100.0%)	0.493
Asymmetry in radiological findings	10 (71.4%)	8 (80.0%)	0.63
Rectus femoris relatively spared compared to other quadriceps muscles	13 (92.9%)	8 (80.0%)	0.550
Flexor digitorum profundus abnormality	9 (64.3%)	10 (100.0%)	0.053
Flexor digitorum profundus more affected than finger extensors	7 (50.0%)	9 (90.0%)	0.079
Mean number of muscles with fatty infiltration per patient	24.6 (17.7)	37.9 (12.0)	0.057
Mean number of muscles with edema per patient	11.3 (12.0)	17.5 (7.9)	0.120

a Mean (standard deviation); *n* (%).

b Wilcoxon’s rank sum test; chi-square; Fisher’s exact test.

## Discussion

The results of this study showed IM-Mito as a clinically heterogeneous condition but demonstrating some muscle imaging similarities with IBM. To date, few observational studies have been published related explicitly to IM-Mito, and only one of them has reported muscle MRI evaluation (3–9, 16). This paper brings additional epidemiological, clinical, and muscle imaging information about IM-Mito compared to IBM.

Although the objective of this study was not to evaluate both conditions from a histological point of view, it was possible to observe that the IBM group presented more dystrophic changes compared to the IM-Mito group. At the same time, inflammatory findings were similar, with many predominantly CD8+ lymphocytic infiltrates of endomysial location and high expression of MHC-I. On the other hand, a greater number of IBM biopsies showed positive aggregates for the degenerative markers TDP-43 and p62.

The mean age of our participants with IM-Mito was 56.9 years, presenting a relatively similar value to those found in literature, which ranges from 45.5 years to 69.5 years (3–9). Like the previous series, most patients with IM-Mito in our study were female (71.4%). Other publications identified female prevalence in IM-Mito varying from 62 to 72% (5, 9).

In our study, patients with IM-Mito exhibited a more diverse phenotype than those diagnosed with IBM. Patients with IM-Mito presented either with dynamic symptoms such as myalgia, proximally predominant weakness, prominent muscle weakness of the quadriceps and flexors of the fingers, or even diffuse muscle weakness. On the other hand, patients with IBM showed significantly more prominent quadriceps or finger flexor muscle weakness when compared to individuals with IM-Mito. A greater presence of early falls and fist sign in patients with IBM also reinforces the preponderance of quadriceps and finger flexor muscle involvement in these cases. Blume et al. (3) described that those patients with IM-Mito had proximal muscle weakness predominantly in the quadriceps. Temiz et al. (5) reported that patients with IM-Mito and IBM had selective weakness of the quadriceps and finger flexors more often than polymyositis. Papadimas et al. (7) described predominantly limb-girdle or diffuse weakness pattern in patients with IM-Mito. Kleefeld et al. (9) evidenced a heterogeneous clinical pattern relatively similar to that found in our study in IM-Mito: most participants had proximal weakness phenotype, some cases were oligosymptomatic with dynamic symptoms, and few patients had predominant quadriceps and finger flexor muscles weakness. As previously mentioned, we also evidenced an oligosymptomatic phenotype with predominant dynamic symptoms such as myalgia, cramps and fatigue in IM-Mito. We particularly observed that myalgia was significantly more frequent in patients with IM-Mito compared to IBM. Temiz et al. (5) had already noted a higher prevalence of myalgia in patients with IM-Mito.

Our results showed some similar radiological muscle MRI features in patients with IM-Mito and IBM. Most of the muscles evaluated showed comparable presence of edema or fatty infiltration in patients with IM-Mito and IBM. Our study showed a trend towards more muscles being affected by fatty infiltration in patients with IBM compared to IM-Mito. Similarly, a previous study evaluating WBMRI showed that the average mean degree of fatty infiltration and proportion of affected muscles per patient appeared to be higher in IBM compared to IM-Mito (16). Muscles with the highest mean degree of fatty infiltration were quadriceps and medial gastrocnemius in our study’s IM-Mito and IBM groups. It is relevant to mention that distal involvement of the vastus intermedius and medial quadriceps muscles on MRI is considered a typical imaging pattern of IBM (15, 22, 23). Additionally, several publications report medial gastrocnemius as the most consistently affected leg muscle in patients with IBM (23–27). In a study that evaluated WBMRI of seven patients with IM-Mito, the authors described high variability in the pattern of muscular involvement regarding fatty infiltration, and they did not find resemblance to IBM imaging features (16). Most of our participants with IM-Mito and IBM demonstrated an imaging pattern of relatively spared rectus femoris muscle compared to other quadriceps muscles. Cox et al. (27) and Phillips et al. (25), in two MRI studies evaluating 50 patients with IBM, also reported relatively spared rectus femoris compared to other quadriceps muscles (25, 27). The previous study evaluating WBMRI in patients with IM-Mito did not specifically mention the finding of relatively spared rectus femoris muscle compared to other quadriceps muscles (16). The most affected upper limb muscle in our series was the flexor digitorum profundus in both IM-Mito and IBM. Guimarães et al. (15), in a study with MRI of 12 patients diagnosed with IBM, also found deep finger flexors to be the most affected muscles in the forearm, being abnormal in 83% of the individuals. Other case series also highlight upper limbs distal involvement preferentially of the flexor digitorum profundus muscle in MRI of patients with IBM (25, 27, 28). Zierer et al. (16) reported a lack of relevant muscle MRI involvement in upper extremities in IM-Mito patients that was attributed to technical reasons related to MRI acquisition procedures. Additionally, our study observed asymmetric radiological involvement in most cases of IM-Mito and IBM. Dion et al. also found asymmetric radiological fatty replacement significantly more frequent in patients with IBM when compared to IM in a study of 50 patients (29). In the study of Zierer et al. (16), asymmetry was commonly observed in patients with IBM but also in IM-Mito and other muscular inflammatory conditions, suggesting low specificity of this finding. Most of our patients with IM-Mito or IBM had a greater mean number of muscles with fatty infiltration than edema. This radiological pattern of greater relative presence of fatty replacement compared to edema on muscle MRI has been reported previously in IBM (30).

Our results also showed some trends towards differences in WBMRI in patients with IM-Mito and IBM. When compared to IM-Mito, there was a tendency for individuals with IBM to have a greater mean number of muscles with fatty infiltration and greater involvement of the flexor digitorum profundus muscle, even though disease duration was similar in both groups. Likewise, a previous study evaluating WBMRI showed that the mean degree of fatty infiltration and proportion of affected muscles per patient appeared to be higher in IBM compared to IM-Mito (16). We also point out that individuals with IBM showed greater presence of fatty infiltration in biceps femoris, semimembranosus, gracilis, tensor fascia lata, and lateral gastrocnemius muscles when compared to individuals with IM-Mito. On the other hand, only vastus medialis muscle showed a greater presence of edema in patients with IBM than in those with IM-Mito.

As mentioned above, our WBMRI findings showed similarities between patients with IM-Mito and IBM. On the other hand, Zierer et al. (16) showed that muscle MRI findings in patients with IM-Mito relevantly differed from IBM. Some methodological aspects may explain these apparently distinct results. From the pathological standpoint, those authors included in the IM-Mito group patients with age-exceeding COX-negative muscle fibers while we chose to select participants with the presence of 1% or more COX-fibers, following other previous publications (5, 7, 8). There was a significant mean disease duration distinction between our patients with IM-Mito (81.9 months) and those included by Zierer et al. (16) (36 months), which may have contributed at least partially to radiological differences found. Additionally, the average age in individuals with IM-Mito was also relatively distinct between our patient population (56.9 years old) when compared to the study by Zierer et al. (16) (64 years old). Regarding semiquantitative radiological scales to measure fatty/fibrous degeneration, those authors utilized the model described by Fischer et al. (31) while we used that published by Mercuri et al. (19). While our study included qualitative and semiquantitative WBMRI evaluation, Zierer et al. (16) also performed quantitative muscle MRI techniques, which have been used to evaluate myopathies including IBM, and have been described as more sensitive for diagnostic purposes (32). We also included more patients classified with IM-Mito (*n* = 14) when compared to those authors (*n* = 7). It should be noted that Zierer et al. (16) mentioned that a small subset of IM-Mito patients showed MRI features that were observed in IBM, but they do not specify the exact number of these individuals.

Our study presents some limitations that should be considered. Diagnostic criteria for patients with IM-Mito is not well determined in the medical literature. There is controversy regarding the cutoff number of COX-fibers that define this condition. Since IM-Mito is a rare condition with little data available so far, our choice was to follow previous publications that included patients with more than 1% COX-fibers to increase diagnostic sensitivity (5, 7, 8). However, other authors used as inclusion criteria for IM-Mito presence of more than 3% of COX-fibers (9), which may increase diagnostic specificity. On the other hand, most patients with IM-Mito in our cohort do not meet the European Neuromuscular Center Criteria for probable or definite IBM. Although the presence of rimmed vacuoles is a classic finding in IBM, there are several descriptions in which this finding may be absent, depending on the muscle biopsied and the stage of the disease (33, 34). Therefore, it is not possible to determine whether cases classified in this study as IM-Mito were IBM based only on the absence of rimmed vacuoles in the muscle biopsy. Additionally, it should be noted that muscle biopsies in our study were performed according to clinical judgment in different muscles (biceps brachii or quadriceps femoris muscles), which may have affected patient selection. Furthermore, muscle biopsies from patients with IBM usually show varying degrees of mitochondrial changes, as observed in our cases (35). The cross-sectional characteristic of our study also limits the power to better characterize IM-Mito from a clinical, radiological, and pathological point of view. In the largest published case series to date with 25 patients with IM-Mito, Kleefeldt et al. (9) found that up to 93% of patients with IM-Mito evolved to IBM over time. These authors even suggest that IM-Mito belongs to the IBM spectrum, and IM-Mito could even be classified as an early form of IBM. Our muscle imaging findings showing similarities in patients with IM-Mito and IBM may help to support this hypothesis, but the reduced sample size and qualitative nature of the evaluation are limitations that make it premature to establish definite conclusions based solely on radiological data. We also did not include another control group with patients diagnosed with polymyositis in our study. Temiz et al. (5) included patients with polymyositis, concluding that individuals with IM-Mito were older, had more selective quadriceps weakness, lower serum creatine kinase, and worse response to immunosuppression when compared to polymyositis.

Our study adds a significant number of IM-Mito patients to previously published literature, revealing a heterogeneous clinical spectrum of this condition. These patients can present with proximal limb-girdle muscle weakness, selective muscle involvement of quadriceps and finger flexors, or even oligosymptomatic. Although the results suggest some similarities in muscle imaging characteristics between IM-Mito and IBM, it remains uncertain whether these two conditions are part of the same clinical spectrum.

## Data availability statement

The raw data supporting the conclusions of this article will be made available by the authors, without undue reservation.

## Ethics statement

The studies involving humans were approved by Ethics Committee Hospital das Clínicas da Faculdade de Medicina da Universidade de São Paulo (file number 3.460.324, CAAE 93788218.0.3001.0068). The studies were conducted in accordance with the local legislation and institutional requirements. The participants provided their written informed consent to participate in this study.

## Author contributions

WC: Writing – review & editing, Writing – original draft, Methodology, Investigation, Formal analysis, Data curation, Conceptualization. AS: Writing – review & editing, Supervision, Investigation, Conceptualization. RM: Writing – review & editing, Supervision, Investigation. CM: Writing – review & editing, Supervision, Investigation. BP: Writing – review & editing. JG: Writing – review & editing, Writing – original draft, Supervision, Methodology, Formal analysis, Data curation, Conceptualization. AO: Writing – review & editing, Investigation, Formal analysis, Data curation. EZ: Writing – review & editing, Writing – original draft, Validation, Supervision, Resources, Project administration, Methodology, Investigation, Funding acquisition, Formal analysis, Data curation, Conceptualization.
